# Overview of Emerging Contaminants and Associated Human Health Effects

**DOI:** 10.1155/2015/404796

**Published:** 2015-12-02

**Authors:** Meng Lei, Lun Zhang, Jianjun Lei, Liang Zong, Jiahui Li, Zheng Wu, Zheng Wang

**Affiliations:** Department of Hepatobiliary Surgery, First Affiliated Hospital of Medical College, Xi'an Jiaotong University, Xi'an, Shaanxi 710061, China

## Abstract

In recent decades, because of significant progress in the analysis and detection of trace pollutants, emerging contaminants have been discovered and quantified in living beings and diverse environmental substances; however, the adverse effects of environmental exposure on the general population are largely unknown. This review summarizes the conclusions of the comprehensive epidemic literature and representative case reports relevant to emerging contaminants and the human body to address concerns about potential harmful health effects in the general population. The most prevalent emerging contaminants include perfluorinated compounds, water disinfection byproducts, gasoline additives, manufactured nanomaterials, human and veterinary pharmaceuticals, and UV-filters. Rare but statistically meaningful connections have been reported for a number of contaminants and cancer and reproductive risks. Because of contradictions in the outcomes of some investigations and the limited number of articles, no significant conclusions regarding the relationship between adverse effects on humans and extents of exposure can be drawn at this time. Here, we report that the current evidence is not conclusive and comprehensive and suggest prospective cohort studies in the future to evaluate the associations between human health outcomes and emerging environmental contaminants.

## 1. Introduction

Emerging contaminants are chemical substances or compounds characterized by a perceived or veridical threat to the environment or human health with a lack of published health criteria. An “emerging” contaminant may also be identified from an unknown source, a new exposure to humans, or a novel detection approach or technology [1, 2]. Emerging contaminants include an extensive array of synthetic chemicals in global use, such as perfluorinated compounds, water disinfection byproducts, gasoline additives, pharmaceuticals, man-made nanomaterials, and UV-filters, which are significant for the development of modern society [2–5]. Because of their rapidly increasing use in industry, transport, agriculture, and urbanization, these chemicals are entering the environment at increasing levels as hazardous wastes and nonbiodegradable substances [1, 2]. Furthermore, adequate and robust epidemic information on their behavior and fate in the global environment, as well as on human exposure, serum and tissue concentrations, and threats to ecological and human health, have not been well documented [2]. Therefore, this review emphasizes the current consensus and representative studies in the relevant fields. Here, we will discuss some emerging contaminants arousing general concern and summarize the evidence with respect to concepts, classification, and application and, particularly, outline potential human adverse effects based on a number of comprehensive epidemic literature reports and representative case reports.

## 2. Perfluorinated Compounds 

Perfluorinated compounds (PFCs), which have been produced since the late 1940s, are composed of a fully fluorinated hydrophobic alkyl chain attached to a hydrophilic end group [6]. Perfluorooctanesulfonate (PFOS), perfluorooctanoic acid (PFOA), and their salts are the most essential representative PFCs and are widely used in fire-fighting foams, lubricants, metal spray plating and detergent products, inks, varnishes, coating formulations (for walls, furniture, carpeting, and food packaging), waxes, and water and oil repellents for leather, paper, and textiles [6–8]. PFCs exhibit high heat, light, and chemical stability, and they are not easily degraded by microbial metabolism [8]. Therefore, PFCs are regarded as persistent, bioaccumulative, and potentially hazardous to animals and humans [7, 9]; however, their transport pathways and global fate have not been adequately documented to date [9, 10]. PFCs undergo wide transportation across all environmental media, including direct sources, such as the production, use, and disposal of consumer products containing these compounds, and indirect sources, such as volatile and neutral PFC precursor degradation [9, 11]. Currently, PFOA and PFOS have been detected in the surface water, sea, wildlife and drinking water, human serum, and even breast milk [7]. According to a study on PFCs, the presence of these species in serum, food, indoor substances, and consumer products and occupational exposure contribute to PFC exposure [12]. Nevertheless, inadequate and limited data about the adverse effects on humans exposed to the environment are available. The outcomes of some investigations on the impact of PFCs and their human health effects are summarized below.

### 2.1. PFCs and Cancer

The potential carcinogenicity of PFOS and PFOA has been investigated in laboratory animals, which demonstrated that these chemicals induce benign liver adenomas, pancreatic adenocarcinoma, and Leydig cell adenomas in rodent models [13–15]; however, the data on PFCs' potential carcinogenicity in the general population are sparse [14, 16]. During 1993 to 2006, Eriksen et al. performed a nested prospective cohort study among 57,053 participants from the general population aged 50–65 with no prior cancer in Denmark to analyze the connection between PFC serum levels and cancer hazard [17]. Considering PFOS and PFOA in the blood plasma as the exposure measurement, the end point was a clinical diagnosis of liver, bladder, prostate, or pancreas cancer during the follow-up duration. The study identified 67, 128, 322, and 713 patients with liver cancer, pancreatic cancer, bladder cancer, and prostate cancer, respectively. Then, a random comparison of 680 men and 92 women without cancer was performed to balance the novel confounded factors for cancer, and the result did not imply a virtual association between PFC concentration and cancer risk, except for prostate cancer in a population in Denmark. That study measured the plasma level of PFCs using only one method. Moreover, the half-life of PFOS varies between 8.7 years and 139 days in humans, and a single measurement of the PFC plasma level may not adequately reflect the plasma levels from previous years or the subject's exposure, which may vary during the observation [16]. Vassiliadou et al. conducted a cross-sectional study in 2010 to measure the serum PFOA and PFOS levels among 40 hospitalized cancer patients at the St. Savas Anticancer Hospital in Athens [18]. Then, the authors compared the results with 56 healthy working employees in an urban area and 86 ambulatory patients and healthy individuals in a rural area; both groups underwent a medical examination in Athens. The outcome showed no significant differences between the three participating groups, except a remarkable difference between the PFOA and PFOS levels of men and women in all groups involved. Because the samples in the study were collected during 2009, the temporal trends of PFOA and PFOS in Greece and any differences between cancer patients and PFCs in the rough cross-sectional data were difficult to assess. Two other case-control studies also investigated PFOA and PFOS levels in the blood of Inuit women with breast cancer (BC) and prostate cancer patients [19, 20]. Bonefeld-Jorgensen et al. used case-control studies to analyze the association between the serum levels of ten types of PFCs in 31 Inuit BC cases and 115 population-based controls without BC [19]. The authors concluded that the serum PFCs might be risk factors for BC in Inuits and that a potential mechanism might be hormone disruption related to xenoestrogenic and xenoandrogenic activities, which increase the risk of developing BC. Eight types of PFC serum levels were measured by Hardell et al. among 201 prostate cancer cases and 186 control subjects without prior cancer [20]. In the cancer group, a higher risk was found for hereditary prostate cancer after adjusting for confounded factors.

These four reports were relatively high-quality studies with long follow-up periods; however, only one study utilized a prospective exposure measurement [17]. Additionally, the cross-sectional study did not balance for confounded factors [18], and the two case-control studies investigated only data collected at a single hospital [19, 20], which could not explain the causal relationship between PFCs and cancer. Above all, the four studies focused on the association between PFC serum levels in the general population and cancer patients, which provided evidence for PFCs' adverse effects on humans, despite various drawbacks in the study designs. Only studies made by Eriksen et al. and Hardell et al. showed statistical significant association between PFC concentration and prostate cancer [17, 20]. The remaining studies did not indicate any causal association between PFC and cancer sites. In future studies, a causal interaction and mechanism between environmental PFC exposures and cancer in a general population would be valuable [14].

### 2.2. PFCs and Other Health Effects

The association between the serum level of PFCs and reproductive dysfunction in a general population has been widely studied in numerous reports addressing infertility, breastfeeding, and semen quality in humans. Fei et al. investigated whether exposure to PFCs and the potential hormonal disruptors might increase infertility [21]. The PFOS and PFOA concentrations with common exposure at weeks 4–14 of gestation were measured among 1240 women in developed areas based on the Danish National Birth Cohort (DNBC) program (1996~2002). The adjusted fecundity odds ratios (FORs) for the three highest-exposure quartiles compared with the lowest quartile of PFOS were 0.70, 0.67, and 0.74, and those of PFOA were 0.72, 0.73, and 0.60, respectively. These outcomes indicate that, at the plasma levels observed, PFOS and PFOA exposure may reduce fecundity in the general population; however, the underestimated association might be higher because of the selection bias: only successful pregnancies were included in this study. In addition, Fei et al. studied the association between parental PFC concentrations and the breastfeeding period, also based on the DNBC [22]. The outcome indicated that PFOS might decrease the women's capacity to lactate, except primipara. The association between the PFOS serum level and multiparous women was not as convincing because multiparous women previously breastfed and because the PFOS serum levels could be reduced through excretion. A potential association was investigated between testicular function, semen quality, and perfluoroalkyl acids (PFAAs) by Joensen et al. [23]. The authors classified PFAA levels into 10 degrees and examined the serum concentrations of reproductive hormones under each PFAA degree to evaluate semen quality among 105 Danish men from the general population. The results implied that higher PFAA concentrations were related to fewer-than-normal sperm but not at statistically significant levels. These studies suggest that exposure to PFCs leads to some reproductive dysfunction; however, the association between exposure levels and the degree of dysfunction remains unclear.

Other studies have emphasized the potential associations between the serum PFOA and PFOS concentrations and prevailing thyroid disease. Pirali et al. measured the PFOS and PFOA levels among 28 participants who underwent a thyroid operation for benign diseases (7 for Graves' disease and 15 for multinodular goiters) and malignant thyroid diseases (5 for papillary carcinoma and 1 for follicular carcinoma) to determine whether there was a significant association between the serum and tissue concentrations of PFCs [24]. All thyroid samples from the surgical specimens were examined to determine the PFOS and PFOA levels. PFOS and PFOA were detected in operational and autopsy thyroid tissues. The average PFOA and PFOS levels were 2.0 ng/g and 5.3 ng/g, respectively, in the surgical specimens, similar to the autopsy thyroid from patients with thyroid diseases. In addition, the serum levels of PFOS and PFOA were remarkably higher than those in the relevant surgical specimens. These outcomes do not indicate that PFOS and PFOA are actively condensed in the thyroid. This study did not provide sample recruitment and control information. Melzer et al. analyzed the PFOA and PFOS levels and health conditions based on the National Health and Nutrition Examination Survey (NHANES project), which encompassed 3,974 adults [25]. The authors analyzed the PFC levels during three different periods and the incidence rates of reported thyroid disease and current thyroid dysfunction in women and men. Employing fully adjusted logistic models, the results indicated that higher PFOS and PFOA serum levels were associated with current thyroid disease among a general adult population in the US. No overlap was detected between the NHANES samples, the measured PFC levels, and the samples from people with thyroid hormones measured, which limited the study data. Knox et al. analyzed the data of the C8 Health Project and found that PFOS and PFOA were correlated with considerable increases in serum thyroxine (T4) and decreases in triiodothyronine (T3) uptake in all cases studied [26]. A cross-sectional study conducted by Shrestha et al. investigated the effects of PFCs on thyroid hormone in 87 older adults in New York [27]. The authors concluded that higher serum perfluoroalkyl substances (PFASs) levels were associated with increased fT4 and T4; however, another cohort study performed by Webster et al. showed that PFASs were significantly correlated with TSH and negatively related to fT4 in a population of pregnant women with higher TPOAb in Canada, which occurred in 6 to 10 percent of pregnant women [28]. Considering the results reported by Pirali et al., lower PFC levels in thyroid tissues than in serum exerted harmful effects on the thyroid [24]. And outcomes drawn from 28 participants and deficient statistical power in this study limited their findings. The three studies proposed that higher PFC serum levels might change thyroid hormone levels [26–28]; however, the results of these studies were contradictory in some aspects, such as serum PFC-related increases in T4 and fT4 and decreases in T3 and hypothyroidism. These discrepancies might be caused by population differences in sex, age, region, individual specificity, exposure level, and objectives and the methods used in the study.

Studies have also focused on the potential associations between the serum PFOA and PFOS concentrations and metabolic diseases. Frisbee et al. analyzed data from the C8 Health Program, which allowed the examination of a very large population of 69,030 US residents living near a chemical production facility that released PFOA [29]. The authors advocated that the arithmetic average (SD) serum PFOA and PFOS concentrations among 12476 adolescents and children were 22.7 ng/mL and 69.2 ng/mL, respectively. After adjusting for covariants, PFOA was substantially associated with increased total lipid and low-density lipoprotein cholesterol (LDL-C), and PFOS was considerably related to increased total lipid, LDL-C, and high-density lipoprotein cholesterol (HDL-C). The relationships between PFOA and PFOS and gene modifications during the process of lipid metabolism in humans were investigated for the first time by Fletcher et al. [30]. Employing adjusted linear regression models, the authors concluded that increased copy numbers of lipid mobilization genes were related to PFOS levels and observed decreased copy numbers of lipid-transport genes. The results implied that PFC exposure might lead to a hypercholesterolemic condition, with further adverse influences on human health. Lin et al. detected the effect of PFCs on glucose homeostasis by measuring the perfluorononanoic acid (PFNA) serum levels among 474 teenagers and 969 adults from the NHANES and found that higher serum PFNA levels were related to hyperglycemia and higher *β*-cell activity [31]. The data from the NHANES were also analyzed by Nelson et al. to investigate the associations between lipid, weight, and PFC serum levels [32]. The authors observed that PFOA, PFOS, and PFNA are significantly related to the total lipid and non-high-density cholesterol (NHD-L) levels and negatively related to insulin resistance and weight. Steenland et al. interrogated the association between uric acid and PFCs and concluded that the PFOA serum concentrations were significantly related to a higher rate of hyperuricemia, which is a potential risk factor for hypertension and other cardiovascular diseases [33]. As a result, based on the current studies, there is inadequate evidence to draw scientific conclusions regarding the potential causal adverse effects of PFCs on human metabolic diseases. In addition, the PFC levels in the environment must be related to the exposure concentration in the human body and further health outcomes.

## 3. Disinfection Byproducts

Disinfection chemicals used in swimming pool and drinking water purification are key components shielding humans from water-borne diseases [9, 34]. These chemicals, which are usually oxidizing agents, possess strong chemical activities that not only eliminate pathogenic agents but also react with many deoxidizers [35]. As a result, undesired byproducts are created during disinfection procedures. The widespread and frequent use of these chemicals produces disinfection byproducts (DBPs), particularly chlorinated DBPs (CDBPs) in purified water, and nearly all humans are exposed to these chemicals in developed regions through swimming pools and drinking water [4, 36]. More than six hundred DBPs have been discovered, including iodinated trihalomethanes (THMs), aldehydes, ketones, halomethanes, hydroxy acids, carboxylic acids, alcohols, keto acids, esters, and even nitrosamines (NDMA) [9, 37]. THMs and HAAs are the two major types of halogenated DBPs, accounting for over 80% [34].

### 3.1. DBPs and Cancer

THMs' carcinogenic effects have been confirmed by numerous studies employing laboratory animals, which show that THMs in the drinking water are associated with colorectal tumors, BC, and bladder tumors as a result of nongenetic toxicity [34]. To date, we have not found adequate, causal evidence to support the relationship between cancer and THMs at typical doses in animals. Unfortunately, sparse findings have been observed on the adverse effects on humans upon THM exposure. Villanueva et al. investigated whether bladder cancer was related to THM exposure by oral and respiratory pathways or dermal absorption of water during bathing and swimming [38]. This study enrolled 1,219 subjects and 1,271 matched participants in a case-control study in Spain during 1998–2001. Long-time THM exposure was related to a twofold-higher incidence of bladder cancer, with an OR of 2.10 and 95% CI of 1.09 to 4.02 for mean household THM concentrations of >49 and ≤8 *μ*g/liter. Compared with participants who did not drink chlorinated water, those with THM exposure of ≥35 *μ*g/day by ingestion exhibited an OR of 1.35 and 95% CI of 0.92 to 1.99. The OR for shower or bath duration by THM concentration was 1.83 with 95% CI of 1.17 to 2.87 for the highest compared with the lowest quartile. Swimming in pools was correlated with an OR of 1.57 (95% CI: 1.18–2.09). Bladder cancer was related to long-duration exposure to THMs from chlorinated water typical of the exposure experienced in developed areas. Nevertheless, a large-scale case-control study conducted by Michaud et al. in Spain indicated that water ingestion was negatively related to bladder cancer without considering THM exposure concentrations [39]. Chang et al. also detected whether DBP exposure was relevant to bladder cancer [40]. The authors designed a matched-pair study to analyze the association between the exposure to total trihalomethanes (TTHM) in the drinking water and the mortality rate of bladder cancer among 65 participants in a Taiwanese province. The adjusted ORs of bladder cancer mortality for the municipality's TTHM levels in the drinking water were 1.8 (95% CI: 1.18–2.74) and 2.11 (95% CI: 1.43–3.11), respectively, in the highest and intermediate groups. The outcome of this investigation indicated that there were positive associations between the levels of TTHM in treated water and bladder cancer morality. Salas et al. conducted a case-control study recruiting 559 hospital controls and 548 incident cases to explore potential mechanism between THM exposure and bladder cancer, and the results indicated that THM exposure might be related to DNA methylation [41]; however, evidence regarding the correlation between DBP exposure and cancer was mixed. A case-control study on DBPs and colorectal cancer was integrated by King et al., who divided participants into two groups (more than 35 years of exposure and not more than 10 years of exposure to chlorinated water) and adjusted for confounders. The OR of colon cancer was 1.63 (1.07–2.48) for ≥75 *μ*g/L and OR of rectal cancer was 0.91 (0.55–1.51) for ≥75 *μ*g/L. For males, long-term exposure to DBP showed an excess risk of colon cancer. And females exposed to DBP were not associated with risk of colon cancer. The association between the risk of rectal cancer and participants exposed to DBP was not observed in this study [42]. Rahman et al. recruited King's study and other 12 studies to investigate on DBP and colorectal cancer by a meta-analysis. The authors suggested that, for colorectal cancer, because of the inconsistencies of the outcomes and poor quality of the relevant investigations, we cannot draw any conclusions [43]. Other cancers, such as breast, pancreas, esophagus, lung, kidney, and brain, were interrogated by sporadic studies, from which no meaningful conclusions can be drawn. Additionally, for melanoma and nonmelanoma, leukemia, and skin cancer, no significant correlations with DBP can be confirmed because of the current inadequate evidence [34]. Overall, a small number of sites have been identified by available evidence on the human body as suspected targets, especially the bladder, but drawing crucial conclusions regarding causality is hindered by intrinsic questions such as methodological drawbacks, exposure assessment limitations [38, 40]. Specifically, which DBPs are the most important compounds and their molecular mechanisms in human beings and dose-response relationship remain to be clarified.

### 3.2. DBPs and Other Health Effects

Recently, these byproducts have been suspected as risk factors for infertility, fetal loss, long gestational duration and poor fetal growth, and fetal anomalies, many of which have been interrogated in published records or current studies. Some studies investigated on DBPs in tap water and semen quality and reported a negative influence of DBP exposure on normal sperm concentration and sperm morphology, but not on motility percentage [44, 45]. And these studies indicated that poor sperm quality in humans was not associated with exposure to levels of DBPs near or below regulatory standard and recommended further study [44–46]. Another study analyzed the association between DBPs and menstrual cycle function based on data from a prospective study and suggested that THM exposure might affect ovarian function with decreased cycle length and follicular phase length [47]; Waller et al. conducted a prospective study and concluded an increased risk of spontaneous abortion for women with consumption of five or more glasses per day of cold tap water containing ≥75 *μ*g/liter of total THMs [48]; Hoffman et al. focused on DPBs exposure and fetal growth, which showed no correlation except average residential concentrations above regulatory limits [49]. As for fetal malformation, Agopian et al. analyzed data from the National Birth Defects Prevention Study (NBDPS) delivered during 2000–2007 and found that gastroschisis might be associated with shower length, but not relevant to bath length or shower frequency [50]. Righi et al. did a case-control study in Italy on 1917 different congenital malformations and indicated a higher risk of newborns with renal defects (OR: 3.30; 95% CI: 1.35–8.09), abdominal wall defects (OR: 6.88; 95% CI: 1.67–28.33), and cleft palate (OR: 4.1; 95% CI: 0.98–16.8) when maternal exposure of chlorite level was 700 *μ*g/L. And higher risks of newborns with obstructive urinary defects (OR: 2.88; 95% CI: 1.09–7.63), cleft palate (OR: 9.60; 95% CI: 1.04–88.9), and spinal bifida (OR: 4.94; 95% CI: 1.10–22) were observed at women exposed to chlorate level of 200 *μ*g/L [51]. This outcome may be because most of water disinfectant in Italy is chlorine dioxide.

Despite the large scale of research, no determined evidence exists to support reproductive hazards related to DBP exposure levels, besides slight correlations with some types of congenital malformations [50, 51]. Despite the fact that disinfection produces hundreds of substances in different proportions, in these studies only small numbers of these pertinent pollutants are normally evaluated and measured, which may contribute to the mostly negative outcomes. In addition, relationships with sex, smoking, genetic susceptibility, and other risk factors must be clarified.

## 4. Gasoline Additives

Gasoline encompasses more than five hundred components, such as the known or suspected carcinogenic substances benzene, 1,3-butadiene, and methyl tert-butyl ether (MTBE) [52]. MTBE, the most widely used oxygenated bunker, is diffusely used as a new unleaded petrol additive, particularly in developing districts [53, 54]. MTBE not only enhances the octane additive used in petrol to improve its burning efficiency and decrease carbon monoxide and other hazardous materials, such as ozone and benzene, in automobile exhaust but may also be used to replace tetraethyl lead as an antiknock species [53, 55]. MTBE is a colorless, smelly liquid with limited water solubility (4 g/100 g in water) and easily infiltrates into soil and spreads to the surrounding environment by volatilization [56]. MTBE can also contaminate surface water and groundwater, seriously threatening drinking water sources. Because of its special structure and properties, MTBE has a long half-life in groundwater and is difficult to degrade. MTBE is rapidly absorbed by inhalation exposures [55]. Human also can be exposed to MTBE by dermal absorption and ingestion of contaminated water [56].

Animal studies have revealed that MTBE can lead to testicular, uterine, and kidney cancer and also harm the kidney, immune system, liver, and central nervous system [53–56]. MTBE, listed as a suspected carcinogen, exhibits a potential toxicity on the human body. Symptoms, including sicchasia, headache, and optical and nasal stimulation, have been suspected to be associated with acute exposure to MTBE. Johanson et al. recruited 10 healthy males to measure the adverse effects of MTBE [57]. Subjective ratings (stimulating symptoms, discomfort, and CNS symptoms) and eye (redness, conjunctival harm, blinking frequency, and break-up time of tear film) and nose (summit expiratory flow, aural rhinometry, and phlogistic markers in rhinal lavage) measurements were analyzed. Solvent smell was the only positive rating noted as the exposure level increased. The blocking index number, an index of nasal swelling, was aggravated with exposure duration, but no exposure-response association was found, showing that MTBE was not the decisive factor for this symptom. Joseph and Weiner performed an analytical study and found that the incidences of cough, headache, throat stimulation, hypersensitive rhinitis, upper respiratory communicable disease, sicchasia, dizziness, wheezing, anxiety, otitis media, insomnia, skin rash, palpitations, malaise, and allergy were associated with the MTBE levels in the air [58]. During the winters of 1994-1995 and 1993-1994, the levels of MTBE and the incidence of these symptoms increased, whereas, during the summer, the MTBE levels and the symptom incidence were both relatively low. Wheezing and asthma were particularly increased. Specific health complaints of MTBE exposure have not been reported in those studies.

Occupational exposure of MTBE among workers such as road tanker drivers, garage workers, and gasoline service station attendants had drawn worldwide concern since 1990s, although such exposure does not take place in general population [59–61]. In order to evaluate neuropsychological adverse effects among 101 road tanker drivers, who were exposed to gasoline that contained 10% MTBE, Hakkola and Saarine compared them with 100 milk delivery drivers working in the same district in Finland [60]. After interviewing based on standardized questionnaires, tanker drivers exhibited a higher fatigue score than milk delivery drivers, and 20% of the tanker drivers complained of nausea or headaches. This study did not illuminate specific exposure MTBE of two groups. Vojdani et al. reported that the proportions of abnormal apoptotic cells lymphocytes were higher (26.4 ± 1.8%) in the group exposed to MTBE and benzene polluted water for five to eight years among 60 people than in the unexposed groups (12 ± 1.3%) and so were lymphocyte DNA adducts [59]. The outcomes indicated that long-term exposure to MTBE and benzene could induce genotoxic damage which might signal initiation of carcinogenicity. However, this study did not measure MTBE and benzene levels over the period in polluted water. Moreover, the outcome observed cannot be attributed to either contaminant for exposure to MTBE and benzene was not characterized.

MTBE was once used to dissolve gallstone or remove residual debris in percutaneous transhepatic removal [61, 62]. Leuschner et al. measured blood concentrations of MTBE in patients with cholelithiasis; the peak concentrations of the special exposure were about 1000-fold higher than concentration in workers who were exposed to inhalation [63]. Although MTBE and its metabolite were measured in the urine and blood of these patients and it took several days for elimination, no adverse effects were reported in this study. In other relevant case reports and reviews, adverse effects such as renal failure, coma, intravascular hemolysis, and bile leak were reported after this therapy [64, 65].

MTBE subchronic adverse effects have been reported by many studies, most of which employed mouse models [53]. These studies found that MTBE was associated with cancer [66, 67], adverse neurotoxic effects [68], increased blood-urea nitrogen (BUN) [69], and others. Some authoritative studies focused on MTBE carcinogenicity, and no carcinogenic risk was confirmed because of insufficient evidence in humans and limited evidence in laboratory animals.

## 5. Manufactured Nanomaterials

Manufactured nanomaterials by definition have a particle size of approximately 1–100 nm, and examples include amorphous silicon dioxide (SiO_2_), carbon nanotubes (CNTs), and titanium dioxide (TiO_2_) [70, 71]; these materials are considered to be emerging contaminants [72]. Manufactured nanomaterials are widely used in sunscreen products, agriculture, transport, healthcare, materials, energy, and information technologies [70, 73–75]; however, novel trace methods for examining the relevant residues and nanoscale pollutants have not been established because of their limited production and relatively immature detection techniques [76, 77]. Nanoscale materials will generate physical and chemical properties, such as particular surface effects, small size effects, and quantum effects, which may produce uncertain biohazard effects [70]. The atomic interface of nanomaterials can cover 15% to 50% of the overall surface area, and this structure provides nanomaterials with strong adsorption capacity in the air, water, and soil, which can adsorb toxic gases (NO_2_, SO_2_, and others), toxic heavy metals (copper, lead, mercury, cadmium, and others), and biologically active substances (polycyclic aromatic hydrocarbons, pesticides, microorganisms, proteins, nucleotides, refractory organics, and others) [78, 79]. Other special properties of nanomaterials, such as their catalytic character and superior toughness and strength, make these materials resistant to degradation using chemical and biological methods [70]. Manufactured nanomaterials undergo long-term migration, conversion processes, and complex chemical reactions in the environment while adsorbing various inorganic and organic molecules on their surfaces. As a result, new pollutants are formed.

In animal tests and in vitro assays, manufactured nanomaterials have shown well-defined carcinogenic potentials, especially reproductive and developmental toxicity at high doses [80]. Some scientists have suggested that nanomaterials may be carcinogenic, regardless of their chemical components [81]. Some epidemiological studies in different periods and regions estimated the association between the exposure levels of TiO_2_ and lung cancer in humans, but none have shown statistically significant results [82–84]. Regarding CNTs, no relevant studies have been published in this field to date [85]. A meta-analysis of twenty-eight cohorts and fifteen case-controls by Pelucchi et al. investigated the exposure levels of silica and lung cancer risk and found no significant evidence to support the carcinogenicity of SiO_2_ [86]. Nanomaterial biological safety issues have attracted worldwide interest [70]. To date, the mechanisms underlying the toxicity to humans and animals remain unknown [71]. Therefore, more attention should be paid to the environmental safety of manufactured nanomaterials and to strengthening research related to human health effects.

## 6. Human and Veterinary Pharmaceuticals

Pharmaceuticals are emerging contaminants in the environment because of their increasing applications in humans and animals [87–89]. Some medicines persist in the body after application and exhibit a novel pattern of action [87]. In recent years, because of the continuously increasing amounts of drugs and advanced ultra-trace detection technologies, considerable human and veterinary drugs have been detected in the environment, especially in water [88, 90, 91]. Approximately three thousand different chemicals involved in human medicine, including lipid regulators, anti-inflammatory drugs, analgesics, contraceptives, neuroactive medicine, antibiotics, and beta-blockers, exist [87, 90]. The main pathway through which pharmaceuticals enter the surface water is human intake, followed by subsequent excretion in municipal wastewater, hospitals, pharmaceutical waste, and landfills [90]. The human pharmaceuticals present in sewage are difficult to remove, and, as a result, high levels of medicine are being released in treated effluents. Inputs of pharmaceuticals into the water systems have been reported in rivers, lakes, treated effluents, and groundwater [90, 92]. After long periods of enrichment, high concentrations of drug residues will threaten human health and the ecosystem [91]. Thus, there is increasing widespread concern about the potential influence of pharmaceutical residues in the environment [87]. Additionally, the considerable use of antibiotics has garnered ubiquitous attention regarding the wide and extensive antibiotic resistance of microorganisms [93, 94].

### 6.1. Veterinary Antibiotics (VAs)

VAs are being increasingly used in many regions to protect the health of animals and treat diseases to improve the feed efficiency of livestock, poultry, pets, aquatic animals, silkworms, bees, and so on [92, 95]. VAs are mainly divided into several pharmacological types: antimicrobial, anthelmintic, steroidal and nonsteroidal, anti-inflammatory, antiparasitic, astringent, estrus synchronization, nutritional supplement, and growth promoter [92]. A large number of antibiotics employed in animal food production are inefficiently adsorbed in the animal's gut, and, as a result, almost 30–90% of these drugs are excreted [96]. Moreover, VA additives can be active and converted back to the prototype after excretion [97, 98]. Thus, a considerable percentage of the veterinary antibiotics may spread into the surroundings in bioactive forms, which will cause long-term adverse effects on the soil, water, microorganisms, plants, and animals and finally affect human health through the food chain [92]. The frequent use of VAs has drawn attention about the potential increasing populations of new resistant strains of bacteria. Detected bacterial populations from gut of animals given antibiotics were about five times to be resistant to common antibiotic resistant microbial strains [99]. Esiobu et al. reported a 70% enhancement in resistance to certain antibiotics including streptomycin, penicillin, and tetracycline after using soil manure from animals in a dairy farm [100]. However, no reliable studies have elaborated the relationship between VAs use and human antibiotics resistance and other health outcomes at present.

### 6.2. Human Pharmaceuticals

Drugs for humans are designed to play an active role in the specific metabolic and molecular pathways [90]; however, some also have side effects in humans. Pharmaceuticals in the environment have ecotoxicological effects on some nontarget species that have the same active sites, such as organs, tissues, cells, and active molecules with target species [91]. Recently, numerous studies focusing on the acute toxic effects to aquatic organisms resulting from drug consumption have been reported. Propranolol exhibits strong acute toxicity on benthos and zooplankton, whose lethal dose of 50% (LC_50_) is about 1 mg/L, and fluoxetine toxicity on benthos is stronger than propranolol, whose LC_50_ is less than 0.5 mg/L [101, 102]. Often, human pharmaceuticals acute toxicity is nonspecific, for example, unspecific membrane toxicity by oxidative stress. Moreover, because acute effects levels are about 100–1000 times higher than pharmaceuticals residues detected in the aquatic environment, acute toxicity to aquatic organisms hardly occurs at detected environmental levels [91]. Studies on environmental pharmaceuticals acute toxicity to human have not been reported yet. The reason may be also due to pharmaceuticals environmental levels being relatively at low levels which cannot induce human adverse effects.

Studies on the long-term exposure toxic effects with lower drug doses are relatively limited. Fenske et al. reported that 17*α*-ethinylestradiol (EE2), a widely used oral contraceptive, induced male gonad developmental arrest in zebrafish exposed to man-made EE2 at an environmentally relevant concentration (3 ng/L) in a flow-through system [103]. Previously, researchers suggested that EE2 is present at low levels and is not harmful, but this study found that zebrafish exposed to a typical environmental concentration of EE2 displayed estrogenic effects, such as abnormal phenotype development and reduced reproductive success. Because of the increasing levels of EE2, estradiol, and other estrogenic substances being detected in surface water, animals with chronic exposure to these biological compounds may suffer some potentially toxic effects without obvious signs.

Most human and veterinary pharmaceuticals exist in the environment at low concentrations that do not cause acute toxic effects. However, some organisms are exposed to low doses over long periods during their lifetime, leading to remarkable chronic toxic effects [91]. Studies on long-term and low-dose exposure are more accurate and should directly reflect the ecotoxicological effects of human and veterinary pharmaceuticals. However, current investigations are not sufficient to derive an accurate profile of the possible hazards of pharmaceuticals, and some studies on animals, such as fish, may imply potential mechanisms and influences on human.

## 7. Sunscreens/Ultraviolet Filters

Sunscreens/ultraviolet filters (UV-filters) are mainly used in personal care products, such as lipsticks, perfumes, hairsprays, hair dye and moisturizers, skin care products, shampoos, and makeup, as well as in noncosmetic products, including furniture, plastics, carpets, and washing powder [104, 105]. Sunscreens are popular protective products against ultraviolet radiation hazards, early skin aging, and skin cancer [106]. UV-filter formulations can be organic (chemicals) or inorganic (minerals) [107]. According to the FDA, inorganic sunscreen scatters UV radiation with wavelengths of 290 to 400 nm [108, 109]. Organic sunscreens absorb novel photons of UV and include 3-(4-methylbenzylidene)camphor (4-MBC), benzophenone-3 (BP-3), 2-ethylhexyl 4-methoxycinnamate (OMC), 2-ethylhexyl 4-dimethylaminobenzoate (OD-PABA), 3-benzylidene camphor (3-BC), homosalate (HMS), and 4-aminobenzoic acid (PABA) [105]. In addition, highly produced lipophilic sunscreens can spread into the aquatic environment by bathing, washing clothes, and swimming [110]. Potential exposure patterns of humans and animals overlap through the food chain.

Many in vitro and in vivo studies based on lab animals have indicated the endocrine-disrupting influences of sunscreen, including disruption of the hypothalamic-pituitary-thyroid axis (HPT) and reproductive and developmental function [105]. Few human investigations have addressed the possible adverse effects of UV-filters, and the study durations are inadequate to be conclusive. Frederiksen et al. investigated BP-3, a UV-filter, and found that it could be detected in 96% of American urine specimens and 85% of Swiss breast milk specimens analyzed [104]; however, UV-filter potential hazards on humans are difficult to assess using exposure data alone. At present, most case reports are related to dermatitis caused by sunscreens [106]. The associations between sunscreens and adverse effects on humans have not been deeply and widely investigated.

## 8. Conclusions

**Figure 1:**
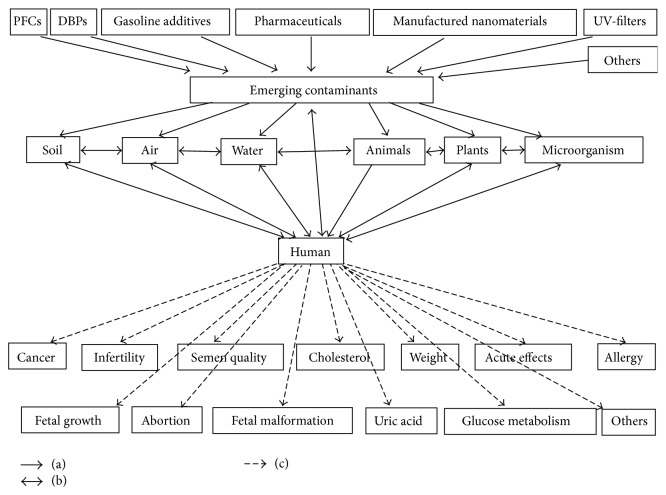
(a) represents that perfluorinated compounds, water disinfection byproducts, gasoline additives, manufactured nanomaterials, human and veterinary pharmaceuticals, UV-filters, and other pollutants are emerging contaminants. The impact of emerging contaminants acts on soil, air, water, animals, plants, microorganisms, and human. (b) represents interactions between soil, air, water, animals, plants, microorganism, human, and emerging contaminants. (c) represents that human in exposure of emerging contaminants may have potential adverse effects.

Humans and the ecosystem as a whole are exposed to various emerging contaminants through different methods, both known and unknown [3, 4]. Our review demonstrates that these contaminants continuously produce emerging and urgent challenges to the soil, water, air, and ecosystems, particularly human health (Figure 1) [2]. Moreover, new chemical outputs spread and usually exceed the abilities of safety remediation, risk evaluation methods, monitoring techniques, and current preventative factors [1, 2]. Given the current conditions, the following several interactive factors should be considered to assess human health effects of pollutant exposure: contaminant concentration, category and properties, scale and level of pollutants, and the hazard intensity generated for health and available resources. The elimination of emerging contaminants in the environment and human body depends on future techniques and studies to establish overall remediation theories and methods [1, 3].

Based on current investigations and studies, the emerging contaminants present suspected mutagenicity, teratogenicity, and carcinogenicity to humans and other animals. On the one hand, no sufficient and large-scale evidence has proven causal associations between emerging contaminants and adverse effects on the human body. On the other hand, whereas the exposure levels in animal experiments may not represent human exposure levels in reality and long-term chronic exposure is seldom employed in animal models, we cannot ignore the adverse effects indicated by animal experiments. Further large-scale studies with improved designs are needed to provide more convincing and clear outcomes.
